# Hemorrhagic cystitis induced by JC polyomavirus infection following COVID-19: a case report

**DOI:** 10.1186/s12894-024-01464-1

**Published:** 2024-04-16

**Authors:** Yuanjie Lv, Xiaoping Liu

**Affiliations:** Department of Infection, Hospital of Traditional Chinese Medicine, Xinchang County, No.188 Shijiu Feng Road, Qixing Street, Shaoxing, 312500 China

**Keywords:** JC polyomavirus, Hemorrhagic cystitis, Coronavirus disease 2019, Metagenomic next-generation sequencing

## Abstract

JC polyomavirus (JCPyV) is a human polyomavirus that can establish lifelong persistent infection in the majority of adults. It is typically asymptomatic in immunocompetent individuals. However, there is a risk of developing progressive multifocal leukoencephalopathy (PML) in immunocompromised or immunosuppressed patients. Though JCPyV commonly resides in the kidney-urinary tract, its involvement in urinary system diseases is extremely rare. Here, we reported a case of a 60-year-old male patient with coronavirus disease 2019 (COVID-19) infection who developed hemorrhagic cystitis after receiving treatment with nirmatrelvir 300 mg/ritonavir 100 mg quaque die (QD). Subsequent metagenomic next-generation sequencing (mNGS) confirmed the infection to be caused by JCPyV type 2. Then, human immunoglobulin (PH4) for intravenous injection at a dose of 25 g QD was administered to the patient. Three days later, the hematuria resolved. This case illustrates that in the setting of compromised host immune function, JCPyV is not limited to causing central nervous system diseases but can also exhibit pathogenicity in the urinary system. Moreover, mNGS technology facilitates rapid diagnosis of infectious etiology by clinical practitioners, contributing to precise treatment for patients.

**Supplementary Information:**

The online version contains supplementary material available at 10.1186/s12894-024-01464-1.

## Introduction

Coronavirus disease 2019 (COVID-19) is an illness caused by the novel coronavirus SARS-CoV-2 and has resulted in a global pandemic with over 700 million confirmed cases and approximately 7 million deaths [1]. Studies have found that patients with COVID-19 infection exhibit both elevated pro-inflammatory mediators and significant immune suppression, which may be attributed to immune dysregulation in response to the infection [2]. In the state of immunological derangement, polyomavirus can transition from a latent phase to a pathogenic phase. Among the known human polyomaviruses, the most extensively studied are BK polyomavirus (BKPyV) and JC polyomavirus (JCPyV). These are currently the only two viruses associated with extracellular vesicles [3–5]. Both BKPyV and JCPyV establish persistent infections in the kidneys, but only BKPyV is typically pathogenic at this site, leading to hemorrhagic cystitis and nephropathy [6]. JCPyV is not only well-known for causing fatal progressive multifocal leukoencephalopathy (PML) but is also associated with other rare neurological disorders such as JC virus granule-cell neuronopathy, JC virus encephalopathy, and JC virus meningitis [6, 7].

JCPyV possesses a closed circular double-stranded DNA genome with a length of 5130 bp, divided into early and late genes, separated by the non-coding control region (NCCR) containing the replication origin (ORI), promoter, and enhancer elements. The genome encodes six major viral proteins (large T and small T antigens, VP1, VP2, VP3, and agnoprotein), as well as several splice variants of the T antigen [8–10]. The large T antigen is the most crucial protein in JCPyV, participating in the transcription and replication of the viral genome. T proteins drive host cells into the S phase for viral replication, regulate the transcription of host and viral genomes, directly participate in viral DNA replication, and interact with numerous cellular proteins to facilitate these processes [9, 10]. Subsequently, the JCPyV agnoprotein acts as a viral channel protein, participating in the release of progeny viral particles and promoting JCPyV reproduction [11–13].

Research indicates that polyomaviruses may undergo periodic reactivation in both individuals with normal immune function and immunocompromised patients, as evidenced by asymptomatic viral urine shedding. It is noteworthy that compared to BKPyV, which is rarely found in the urine of healthy adults, JCPyV viral shedding is more common and increases with age [14]. A study involving 400 healthy blood donors found that JCPyV viral shedding was significantly more frequent and had higher viral loads compared to BKPyV viral shedding (19% vs. 7%, *P* < 0.0001) [15]. Research suggests that asymptomatic viral shedding may progress to hematuria and develop into symptoms of cystitis as tissue damage increases, with hemorrhagic cystitis being more common with BKPyV infection. JCPyV is a latent infection in the majority of humans, typically persisting in an asymptomatic state [16]. However, in cases of immune dysregulation, JCPyV can be reactivated, transitioning from latency to a pathogenic phase [17, 18]. This study reports, for the first time, a case of immunocompromised patient, due to COVID-19 infection, in whom JCPyV infection was confirmed to be activated leading to hemorrhagic cystitis through metagenomics next-generation sequencing (mNGS) results.

## Case presentation

A 60-year-old male presented to our hospital on May 29, 2023, complaining of fever and sore throat for two days. Upon admission, the nucleic acid test for the novel coronavirus was positive, leading to the consideration of hospitalization in the Infectious Pulmonary Disease Department for the treatment of COVID-19 infection. Twenty-four years prior (Surgery performed in 1999), the patient underwent mitral valve and tricuspid valve replacement, as well as pulmonary valve repair. He had been receiving long-term oral anticoagulation therapy with warfarin 2.5 mg quaque die (QD). Additionally, he had an 8-year history of hypertension (Diagnosed in 2015). On the day of admission, the patient complained of low-grade fever, sore throat, fatigue, and poor appetite, without symptoms such as chest tightness, shortness of breath, abdominal pain, diarrhea, urinary frequency, urgency, dysuria, or hematuria. Physical examination revealed no positive signs. Blood tests upon admission showed a white blood cell count of 12.7 × 10^9^/L and a C-reaction protein (CRP) level of 17.9 mg/L, with other parameters within normal limits. Coagulation function tests showed a prothrombin time (PT) of 21.3 s, an activated partial thromboplastin time (APTT) of 44.5 s, and an international normalized ratio (INR) of 1.82R. Urinalysis, cardiac enzyme panel, renal function, liver function, and other indicators were normal. Chest CT showed chronic inflammation in both lungs. The patient was started on nirmatrelvir 300 mg/ritonavir 100 mg QD and methylprednisolone injection 40 mg QD for anti-COVID-19 treatment while continuing the anticoagulation therapy with warfarin 2.5 mg QD. With the anti-COVID-19 treatment, the patient’s body temperature gradually normalized, and symptoms improved. On the 5^th^ day (June 2, 2023) of hospitalization, nirmatrelvir/ritonavir and methylprednisolone were discontinued.

On the 8^th^ day (June 5, 2023) of hospitalization, the patient suddenly developed hematuria, prompting discontinuation of warfarin. On the 9^th^ day (June 6, 2023) of hospitalization, urinalysis revealed 3 + occult blood, with a red blood cell count of 1022 cells/μL and a white blood cell count of 5 cells/μL. Blood tests showed a white blood cell count of 10.4 × 10^9^/L and a CRP level of 34.57 mg/L, with other parameters within normal limits. Coagulation function tests showed a PT of 17.8 s, an APTT of 43.6 s, and an INR of 1.51R. Renal, ureteral, bladder and prostate ultrasound revealed no abnormalities. A possible urinary tract infection was considered. Therefore, on the 9^th^ day (June 6, 2023) of hospitalization, the patient was started on levofloxacin tablets 0.5 g QD for antimicrobial therapy. However, the treatment response was poor, with the gradually worsened hematuria symptoms and intermittent low-grade fever (around 37.5 ℃). On the 11^th^ day (June 8, 2023) of hospitalization, the urinalysis was counterchecked, showing 3 + occult blood, a red blood cell count of 19,502 cells/μL, and a white blood cell count of 152 cells/μL. Repeated blood routine examination showed a white blood cell count of 10.6 × 10^9^/L and a CRP level of 16.7 mg/L, with other parameters within normal limits. Coagulation function tests demonstrated a PT of 16.4 s, an APTT of 36.4 s, and an INR of 1.39R. Renal function, liver function, and cardiac enzyme panel were normal.

**Fig. 1:**
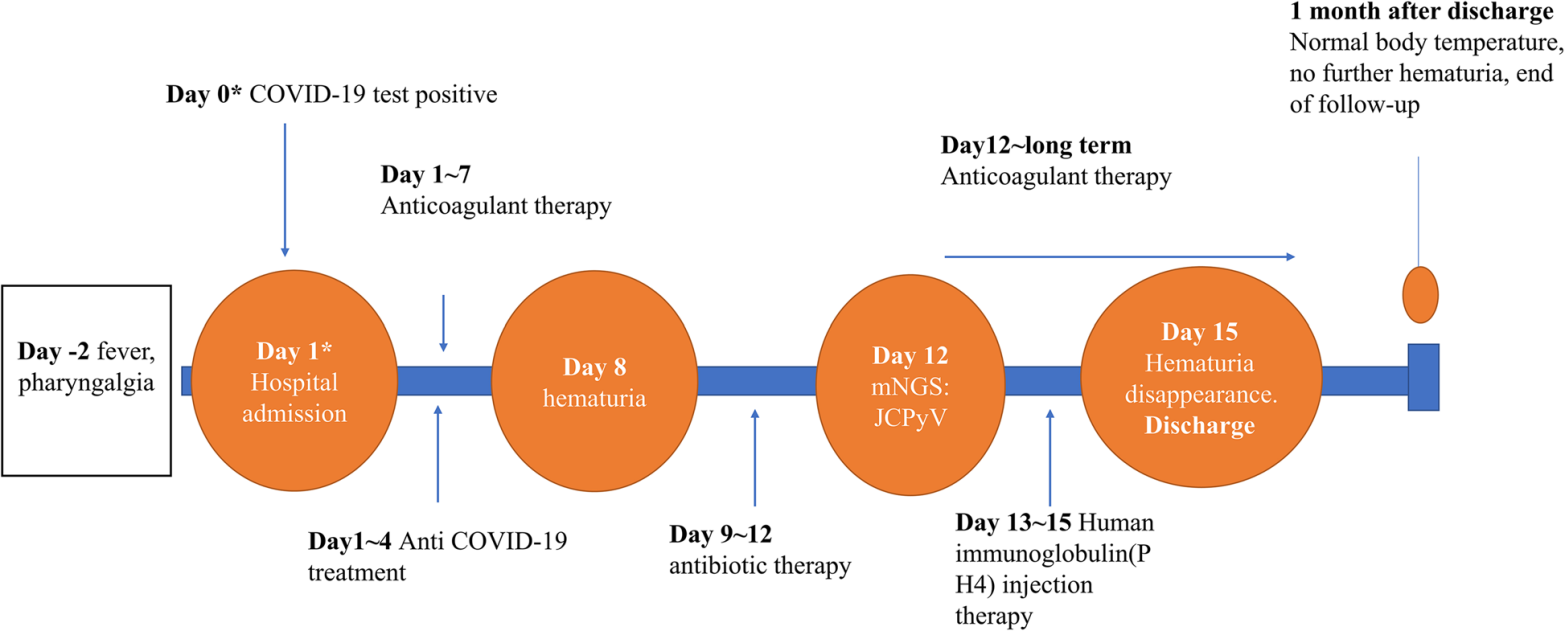
Timeline of significant events before and after patient admission. *Day 0: COVID-19 testing was conducted, with a positive result. The patient was admitted for treatment on the same day (Day 1). Therefore, Day 0 and Day 1 refer to the same day

The urine culture results were negative, and we could not identify the cause of hematuria. We recognize that this may be due to limitations in laboratory testing conditions, making it challenging to detect the pathogenic microorganisms responsible. To expedite the diagnosis, on the 12^th^ day (June 9, 2023) of hospitalization, a midstream urine sample from the patient was sent to a third-party clinical laboratory (Zhejiang Luoxi Medical Laboratory Co., Ltd) for mNGS testing. Details of the mNGS method and quality control are provided in Supplementary file 1. The results revealed JCPyV type 2 with a sequence count of 919 and a relative abundance of 96.84%. We considered the patient to have hemorrhagic cystitis caused by JCPyV, unrelated to the treatment of warfarin. Consequently, we reinitiated the patient’s warfarin therapy at a dose of 2.5 mg QD and discontinued levofloxacin. Starting from the 13^th^ day (June 10, 2023) of hospitalization, human immunoglobulin (PH4) for intravenous injection at a dosage of 25 g QD was commenced to treat hemorrhagic cystitis induced by JCPyV infection. After 3 days (June 10–12) of treatment, the patient’s body temperature normalized, and there was no further hematuria, leading to discontinuation of the medication. The patient was discharged on the 15^th^ day (June 12, 2023) of hospitalization and continued long-term anticoagulation therapy with warfarin at a dose of 2.5 mg QD (for anticoagulation following previous cardiac valve replacement surgery). One month after discharge, the patient had a follow-up visit at the outpatient clinic. Urinalysis revealed a presence of 56 fungi/μl. Considering that the patient had a normal body temperature and no signs of urinary tract infection such as hematuria, chills, or febrile episodes, the result was considered a possible contamination of the urine specimen. Therefore, no treatment was administered. The follow-up was concluded (Fig. 1).

## Discussion

JCPyV is a non-enveloped DNA virus with a seroprevalence of 50% to 70% in adults [15, 19, 20]. As a conditional pathogen, JCPyV virus can cause various diseases in the central nervous system, with the most prominent being PML, a demyelinating disease involving lytic infection of glial cells [21–23]. The prerequisite for PML is profound suppression of cell-mediated immunity, whether disease-related, such as with HIV or lymphoproliferative malignancies, or due to immunosuppressive or immunomodulatory therapies (multiple sclerosis or rheumatoid arthritis) or a combination of both (systemic lupus erythematosus) [21]. Additionally, JCPyV can infect meningeal and choroid plexus cells, leading to JCPyV meningitis (JCVM) [24]. Studies have found that JCPyV VP1 capsid protein deletion mutants contribute to facilitating JCV entry or replication into granule cell neurons, causing JC virus granule cell neuronopathy (JCV GCN) [25, 26]. The fulminant JCPyV encephalopathy (JCE) can also involve cortical pyramidal neurons, characterized by cortical gray matter infection and dissolution [27]. Furthermore, after the initial infection, JCPyV can remain latent in different sites, such as B cells, the brain, spleen, and notably, the urogenital tract (as the most epidemiologically relevant latent site) [14].

The JCPyV virus undergoes persistent latent infection in the kidneys and is shed into the urine [28]. In healthy individuals, the urinary shedding of JCPyV is usually asymptomatic and intermittent, with a higher prevalence and a higher viral load compared to BKPyV [15]. Conversely, in immunocompromised patients, BKPyV urinary shedding is more common [29]. Despite the high viral load in urine, most patients remain asymptomatic, suggesting effective compensatory mechanisms in the cytopathic loss of urothelial cells. Notably, evident diseases induced by JCPyV are observed only under specific circumstances. For example, approximately 50% of kidney transplant recipients develop Polyomavirus-associated nephropathy (PVAN) caused by BKPyV, ultimately leading to graft failure [30, 31]. BKPyV-induced hemorrhagic cystitis occurs in 57% of bone marrow transplant recipients [32]. Interestingly, we observed that JCPyV-induced kidney disease is rare. JCPyV-related nephropathy is a severe but extremely rare complication in kidney transplant recipients, with an incidence rate of only 0.9% in a cohort of 103 kidney transplant recipients [28, 33]. Despite the close homology between BKPyV and JCPyV, there are significant biological differences in their pathogenesis [34].

Although persistent microscopic hematuria caused by JCPyV infection has been reported by Chiarinotti D et al. [35] and Di Maida F et al. [36], as well as JCPyV-induced PVAN being reported [37, 38], these instances are limited to individual case reports. Unlike the more common urological manifestations caused by BKPyV, cases of hemorrhagic cystitis induced by JCPyV are exceptionally rare. JCPyV, as a conditional pathogenic agent, rarely causes hemorrhagic cystitis. Recent literature suggests that although replication of JCPyV in the urinary tract is common, it is associated with a lower incidence of renal disease, indicating that JCPyV viruria may be a protective factor against kidney diseases [39, 40]. It is believed that the long-term coevolution of human polyomaviruses with the host has led to the loss of their pathogenicity, while unique selective pressures encountered in immunocompromised hosts have driven host intramolecular evolution, resulting in the emergence of pathogenic polyomaviruses [41]. In other words, changes in the host’s immune status can reactivate the virus and cause significant pathological reactions in a few cases. This may partially explain the occurrence of JCPyV-associated hemorrhagic cystitis in the case we reported, who had recently experienced a COVID-19 infection prior to the onset of hematuria. It is well known that COVID-19 infection disrupts the host immune homeostasis and stimulates an excessive inflammatory response [42], with severe cases even experiencing a “cytokine storm”, leading to severe clinical complications [43]. In the elderly population, the severity of COVID-19 is exacerbated due to underlying comorbidities and immune senescence [44]. The case we reported involved a 60-year-old patient with hypertension and a history of heart valve replacement surgery, with an elevated CRP index above the normal range (> 10 mg/L) after admission. CRP is a major acute-phase protein [45], and its levels increase in response to injury, infection, and inflammation [46]. In our report, there was an increase in CRP levels (34.57 mg/L vs 17.9 mg/L) during the occurrence of hematuria, indicating an exacerbation of systemic infection or inflammation. This indirectly suggests that the occurrence of JCPyV-induced hemorrhagic cystitis in this case may be related to the disruption of host immune homeostasis following COVID-19 infection, leading to reactivation of JCPyV. However, we did not perform testing for T lymphocyte subtypes, complement, and other immune response molecules, which limits our comprehensive understanding of the specific immune changes in the patient’s body following COVID-19 infection. In addition, an early study in Wuhan, China, reported that among 701 COVID-19 patients, the incidence of acute kidney injury was only 5.1%, while proteinuria (43.9%) and hematuria (26.7%) were very common [47]. In the pathophysiology of COVID-19 infection, ACE2 receptors facilitate intracellular entry and replication of the SARS-CoV-2 virus [48]. Research also suggests a close association between ACE2 receptors and organ damage related to COVID-19 [49]. This may also be one of the reasons for the hematuria observed in this case.

In 2022, a study titled “Seroprevalence of JCV during the SARS COVID-19 Pandemic” assessed the seroprevalence of JCPyV in the first year following the COVID-19 outbreak and compared it with the seroprevalence of previous years. The results indicated a threefold increase in seroprevalence after the pandemic, although statistically nonsignificant, it was notably elevated [50]. The research also found that JCPyV could induce blood coagulation in human O-type red blood cells, fostering seroepidemiological investigations that have led to the global identification of JCPyV. A substantial proportion of the population experiences seroconversion before adulthood [51, 52], and healthy individuals, including pregnant women, can produce immunoglobulin G (IgG) antibodies against JCPyV [53, 54]. However, current seroepidemiological research predominantly focuses on immunocompromised patients with JCPyV reactivation causing PML [55, 56]. Research on JCPyV-induced urological diseases is relatively limited, and future studies should strengthen serological investigations to delve into the underlying pathogenic mechanisms.

In this report, we employed mNGS as a rapid diagnostic tool for JCPyV infection. Conventional diagnostic methods were unable to identify the pathogen in this patient. Our report effectively demonstrated the potential of mNGS to improve the identification of rare pathogenic agents. Previous methods for viral detection have included antigen–antibody analysis [57] and polymerase chain reaction (PCR)-based techniques [58]. mNGS, a recently popularized high-throughput sequencing approach, has been utilized to assist in pathogen detection from various body fluids, such as cerebrospinal fluid, bronchoalveolar lavage fluid, and plasma, offering unique advantages in comprehensive profiling of known and unknown pathogens [59, 60]. Due to its high degree of specialization and cost, mNGS cannot currently be considered a diagnostic tool integrated into routine clinical practice. However, with technological advancements, it is imperative to reduce costs and standardize procedures. It is believed that once mNGS achieves a comprehensive transition from scientific research to clinical practice, it will significantly transform disease diagnosis and treatment approaches.

Currently, there are no specific antiviral drugs or other options available for JCPyV, apart from immune reconstitution [23]. In this report, effective control of hemorrhagic cystitis was achieved by administering human immunoglobulin (PH4) for intravenous injection to the patient for three days. Human immunoglobulin (PH4) for intravenous injection is a therapeutic preparation of normal human Immunoglobulin G (IgG) obtained from healthy blood donors, which can be used as a replacement therapy or an immunomodulator in patients with primary or secondary immunodeficiencies [61]. Antiviral drugs such as chlorpromazine, cytarabine, and topotecan are also widely used in patients with JCPyV-associated diseases [34]. Furthermore, researchers are developing vaccines targeting JCPyV [19], aiming to prevent the development of JCPyV-associated diseases. Recently, the immune checkpoint inhibitor pembrolizumab has been reported to treat a patient with JCPyV-induced PML, demonstrating the great therapeutic potential of anti-PD-1 therapy in PML patients [62]. With the advancement of technology, there will be more treatment options available for JCPyV infection.

In this case, under the condition of immune dysregulation, JCPyV induces the occurrence of hemorrhagic cystitis, challenging the previous understanding that JCPyV in urine is non-pathogenic [63], thus enhancing our understanding of the outcomes of JCPyV infection. Moreover, mNGS technology has been reported as a novel and effective tool for rapidly diagnosing infectious etiologies, emphasizing its potential contribution to precise treatment decisions by clinical practitioners.

### Supplementary Information

**Supplementary Material 1.**

## Data Availability

The data and materials in the current study are available from the corresponding author on reasonable request.
