# Supplementary material for: Hemorrhagic cystitis induced by JC polyomavirus infection following COVID-19: a case report
Source: BMC Urol. 2024 Apr 16;24:87. doi: 10.1186/s12894-024-01464-1 (PMC11020351; doi:10.1186/s12894-024-01464-1)
Supplement: Supplementary file 1 — Supplementary Material 1. [file 12894_2024_1464_MOESM1_ESM.docx]

**Supplementary file 1**

**Procedure and quality control of mNGS**

**1. Procedure of mNGS**

The urine samples are stored in sterile containers and preserved in dry ice for transportation. The entire sequencing and pathogen detection process is conducted in the laboratory of Luoxi Medical Technology Co., Ltd.

(1) DNA from the samples is extracted using the QIAsymphony Circulating NA Kit (Cus.48), with the inclusion of a negative control (RNase-free water) and a positive control.

(2) DNA libraries are prepared using the Illumina TruePrepTM DNA Library Prep Kit V2 (Vazyme Biotech Co., Ltd, TD503).

(3) Sequencing is performed using the Illumina NextSeq 500 system with a 75-cycle kit^1^.

(4) Raw data are quality-controlled using fastp (v0.19.4)^2^. Sequences with a length less than 50 bp, bases with a Phred quality score less than 20, sequences with more than 40% of bases not meeting quality standards, and sequences with more than three “N” bases are filtered out. Processed data are stored in fastq file format on the hard drive.

(5) Burrows-Wheeler Alignment (BWA) is employed to subtract human host sequences mapped to the human reference genome (hg19), generating high-quality sequencing data^3^.

(6) Sequence quality assessment is performed using fastqc (v0.11.5)^4^.

(7) The remaining data after removing low-complexity reads are classified by simultaneously aligning to four microbial genome databases (including viruses, bacteria, fungi, and parasites). Reference databases for classification are downloaded from NCBI (ftp://ftp.ncbi.nlm.nih.gov/genomes/) and Kraken 2 is utilized^5, 6^.

**2. Quality control of procedure**

During the sampling, storage, transportation, and sequencing processes, strict aseptic procedures are followed to ensure the collection of qualified urine samples. The midstream portion of the urine was collected. In the laboratory, specimens are stored in a refrigerator at -80 degrees Celsius to prevent nucleic acid degradation. All testing procedures are completed within 48 hours of receiving the specimen to ensure timely guidance for clinical treatment.

Nucleic acid extraction and library preparation are conducted concurrently with quality control samples. The results of the entire process are compared with those analyzed by the BWA software, and the outcomes of these two procedures were highly consistent. To eliminate background interference, the minimum threshold for reporting the detection of microorganisms was set at 10 RPM-r (RPM defined as reads per million, RPM-r defined as RPMsample/RPMno-template-control).

Base quality score (Q): The Q30 base quality score is a metric used in high-throughput sequencing. For each sequenced base, a corresponding quality score is assigned, reflecting the confidence and error rate in base identification during the sequencing process. These scores are typically represented in ASCII code and are calculated using the formula Q = -10lgP, where P is the probability of an error in base identification. The higher the Q value for a base, the lower the likelihood of identification errors, indicating higher confidence. Q30 specifically indicates that the probability of a base identification error is 0.1%. For next-generation sequencing (mNGS) analysis, the Q30 base ratio is generally expected to be no less than 80%. In this study, the patient’s test report indicates a Q30 value of 94.55%.

References

1. Jeon YJ, Zhou Y, Li Y, Guo Q, Chen J, Quan S, et al. The feasibility study of non-invasive fetal trisomy 18 and 21 detection with semiconductor sequencing platform. PloS one. 2014; 9:e110240.

2. Chen S, Zhou Y, Chen Y, Gu J. fastp: an ultra-fast all-in-one FASTQ preprocessor. Bioinformatics (Oxford, England). 2018; 34:i884-i90.

3. Li H, Durbin R. Fast and accurate short read alignment with Burrows-Wheeler transform. Bioinformatics (Oxford, England). 2009; 25:1754-60.

4. de Sena Brandine G, Smith AD. Falco: high-speed FastQC emulation for quality control of sequencing data. F1000Research. 2019; 8:1874.

5. Wood DE, Lu J, Langmead B. Improved metagenomic analysis with Kraken 2. Genome biology. 2019; 20:257.

6. He Y, Fang K, Shi X, Yang D, Zhao L, Yu W, et al. Enhanced DNA and RNA pathogen detection via metagenomic sequencing in patients with pneumonia. Journal of translational medicine. 2022; 20:195.
